# High Resolution Temperature Measurement of Liquid Stainless Steel Using Hyperspectral Imaging

**DOI:** 10.3390/s17010091

**Published:** 2017-01-05

**Authors:** Wim Devesse, Dieter De Baere, Patrick Guillaume

**Affiliations:** Vrije Universiteit Brussel, Department of Mechanical Engineering, Pleinlaan 2, B-1050 Brussels, Belgium; dieter.de.baere@vub.ac.be (D.D.B.); patrick.guillaume@vub.ac.be (P.G.)

**Keywords:** hyperspectral imaging, temperature measurement, laser material processing

## Abstract

A contactless temperature measurement system is presented based on a hyperspectral line camera that captures the spectra in the visible and near infrared (VNIR) region of a large set of closely spaced points. The measured spectra are used in a nonlinear least squares optimization routine to calculate a one-dimensional temperature profile with high spatial resolution. Measurements of a liquid melt pool of AISI 316L stainless steel show that the system is able to determine the absolute temperatures with an accuracy of 10%. The measurements are made with a spatial resolution of 12 µm/pixel, justifying its use in applications where high temperature measurements with high spatial detail are desired, such as in the laser material processing and additive manufacturing fields.

## 1. Introduction

A good knowledge of the temperature distribution in a part is important in many material processing applications. This is especially true in the fields of laser-based additive manufacturing and laser material processing, including technologies such as laser welding, cutting, hardening and cladding. These techniques all have in common that a high power laser beam is focused onto a metallic part which results in strong local heating and potential melting of the surface. Due to the tight focusing of the laser beam, large temperature gradients exist in the part and the distribution of this temperature field has important consequences for the material properties of the processed part.

In order to control the material properties and geometrical quality of a part, it is important to monitor and possibly regulate the temperature distribution during the process [1,2]. A lot of research is therefore directed towards the design of effective melt pool monitoring methods which can acquire reliable temperature information, possibly to be used in automatic feedback control systems [3,4,5,6,7]. Typical contact temperature sensors are not suitable for melt pool measurements, as the presence of the melt pool creates a destructive environment for these sensors. The melt pool properties themselves would also be disturbed by the sensor, which is why a contactless measurement system is usually preferred.

In the past, multiwavelength pyrometers with one, two or more colours have been used by Doubenskaia et al. [8,9,10], Smurov et al. [11] and Muller et al. [12] to provide point measurements of the temperature in the melt pool during laser welding and laser cladding processes. These methods have the inherent drawback that the variation of emissivity with wavelength needs to be known in order to calculate a temperature value from the measured radiance values [13]. Because this information is usually not available, the radiating object needs to be treated as a grey body, which is not always appropriate and can lead to significant errors, as shown mathematically by Coates [14], Khan et al. [15,16], Gathers [17] and Corwin and Rodenburgh [18]. The use of a pyrometer as a temperature measurement device presents some additional drawbacks such as the difficulty of focusing on a very small spot with a known location and a high sensitivity to noise.

Temperature measurements with high spatial resolution can be made with a camera-based system. Such a camera captures grey scale images that are converted into temperature maps by assigning a fixed value to the emissivity and assuming that this value remains constant throughout the entire image. The emissivity can be found by identifying the melting point in the image, since the value of the temperature is known at this location. Price et al. [19,20] used the region of constant temperature resulting from the phase change of the substrate material to detect the melting point during an electron beam melting process with a Ti6Al4V alloy. The same method was followed by Yadroitsev et al. [21] during a selective laser melting process, which resulted in comparable temperature profiles. Measurements during laser cladding of steel were made by Griffith et al. [22] and Hofmeister et al. [23] using a minimum in the measured temperature gradient for selecting the location of the melting point. A slightly alternative approach was used by Hu and Kovacevic [24] who used visual imaging to locate the boundary of the melt pool.

The temperature profiles obtained with a camera-based system provide useful qualitative information about the change of temperature inside the melt pool, which is not practical to obtain with the use of pyrometers. However, as the emissivity at the melting point may differ substantially from that of the liquid material, no absolute temperature values can be obtained. In addition, the dependence of emissivity with wavelength cannot be taken into account and a grey body assumption is always implicitly made. Finally, the exact value of the temperature at the phase change may not be known: the high heating and cooling rates that are present during these laser material interaction processes could result in a shift of the melting point because the material is no longer in a state of thermodynamic equilibrium [25]. Instead of deriving the emissivity from the melting point, an alternative approach is to measure the emissivity directly using an additional sensor. This was done by Kraus [26,27], who directed a HeNe laser beam to the melt pool and measured its reflectance in order to obtain a value for the spectral directional emissivity at a specific location in the melt pool during stationary gas tungsten arc welding of stainless steel. However, the obtained accuracy of the system is again limited by the requirement of the spatial and spectral independence of the emissivity.

In this paper, the use of a hyperspectral line camera is presented as a means for obtaining spectral radiance measurements at many different wavelengths of a large number of closely spaced points. The temperatures of the points can be deduced from the measured spectra using a suitable curve fitting procedure. An important advantage of this approach is that it significantly increases the signal-to-noise ratio compared to pyrometry methods that use only a small number of wavelengths. Since it is a camera-based system, it also results in a temperature profile of the melt pool surface with a very high spatial resolution. The main advantage compared to classical camera-based systems is that a complete spectrum is available at each location, so that the emissivity can be estimated at each point and the need for a spatially fixed emissivity value is avoided. The spectral dependence of the emissivity is taken into account by allowing it to vary linearly with wavelength. This allows the calculation of an upper and lower bound of a temperature interval that includes the true absolute temperature value.

The concept of hyperspectral imaging has been in use for many years in several different domains [28]. It is widely used in the food industry for quality assurance and sorting applications [29,30] and in the medical field for tissue analysis [31]. Another important domain of application is remote sensing, where it is used as a powerful tool for classification of soil and vegetation [32]. Despite its popularity as a tool for classification and analysis, no report can be found in the literature about the use of hyperspectral imaging as an instrument for high temperature measurements. However, the high spatial and spectral resolution of such a device can be exploited to obtain an accurate temperature distribution, which contains valuable information for many laser material interaction processes.

The paper starts by presenting the mathematical method used for determining the absolute temperature from a number of spectral radiance measurements. The hyperspectral camera is then presented in Section 3 together with an uncertainty analysis of the measurements that can be obtained with this system. The presentation and discussion of the experimental results are given in Section 4, and final remarks and conclusions are formulated in Section 5.

## 2. Temperature Estimation

According to Planck’s law [33], the spectral radiance B emitted by an ideal black body can be written as a function of the wavelength *λ* and temperature *T*:

(1) $$B\;\left(\lambda ,T\right)=\frac{2hc^{2}}{\lambda^{5}}{\left[\exp\;\left(\frac{hc}{k_{B}\lambda T}\right)-1\right]}^{-1}.$$

In this equation, *c* is the speed of light, *h* is the Planck constant and $k_{B}$ is the Boltzmann constant. Multiplying Equation (1) by a spectral emissivity $\varepsilon_{\lambda }$ results in a more general description of the spectral radiance emitted by a real body. This emissivity has a value between 0 and 1 and can be an arbitrary function of wavelength, temperature, material composition and different material properties. Measuring the spectral radiance at a number of uniformly spaced wavelengths $\lambda_{i}$ (i=1,…,N) results in a set of measurements $E_{i}$ that include some additional measurement noise $n_{i}$:

(2) $$E_{i}=\varepsilon_{\lambda }\;\left(\lambda_{i}\right)\;B\;\left(\lambda_{i},T\right)+n_{i}.$$

A frequently made assumption is that the emissivity is wavelength independent in the spectral region of interest. This leads to a so-called grey body description, in which $\varepsilon_{\lambda }$ is equal to a constant value for a fixed measurement location. However, this assumption is not always appropriate. For metals, it can be shown theoretically that the emissivity must be a decreasing function of wavelength for large wavelengths, i.e., in the infrared region [34]. This has been confirmed in numerous experiments and has been shown to hold for stainless steels for smaller wavelengths up to the near infrared and visible region [35,36,37,38]. A more general description of the emissivity can therefore be constructed as a linearly decreasing function of the wavelength:

(3) $$\varepsilon_{\lambda }\;\left(\lambda_{i}\right)=B_{\varepsilon }-A_{\varepsilon }\frac{\lambda_{i}-\lambda_{1}}{\lambda_{N}-\lambda_{1}}\;\;\left(0\leq A_{\varepsilon }<B_{\varepsilon }\leq 1\right).$$

The constraints on the constants $A_{\varepsilon }$ and $B_{\varepsilon }$ result from the requirements that the slope must be negative and that $0<\varepsilon_{\lambda }\leq 1$. The average spectral emissivity value over the measured spectral range will be denoted by *ε* and is given by:

(4) $$\varepsilon =B_{\varepsilon }-\frac{A_{\varepsilon }}{2}.$$

Assuming that the noise $n_{i}$ is zero-mean and Gaussian distributed with known variance $\sigma_{E_{i}}^{2}$, the maximum likelihood estimates of the temperature *T* and emissivity constants $A_{\varepsilon }$ and $B_{\varepsilon }$, given the noisy measurements $E_{i}$, are obtained by solving the following nonlinear least squares problem:

(5) $$\left({\hat{A}}_{\varepsilon },{\hat{B}}_{\varepsilon },\hat{T}\right)=\text{arg}\min_{\left(A_{\varepsilon },\;B_{\varepsilon },\;T\right)}\sum_{i=1}^{N}\frac{{\left|E_{i}-\left(B_{\varepsilon }-A_{\varepsilon }\frac{\lambda_{i}-\lambda_{1}}{\lambda_{N}-\lambda_{1}}\right)B\;\left(\lambda_{i},T\right)\right|}^{2}}{\sigma_{E_{i}}^{2}},$$

subject to the constraints:

(6) $$0\leq A_{\varepsilon }<B_{\varepsilon }\leq 1.$$

In general, this problem has a locally minimizing solution. However, as stated by Coates, there is no guarantee that this solution corresponds to the true temperature: without a perfect knowledge of the spectral emissivity variation, the true temperature value can only be determined within a range bounded by a lower value $T_{l}$ and an upper value $T_{u}$ [14]. These bounds can be estimated by assuming that the emissivity varies linearly according to Equation (3) with an unknown value of the slope $A_{\varepsilon }$. For each possible value of $A_{\varepsilon }$, a combination of $B_{\varepsilon }$ and *T* can be found that minimizes the cost function in Equation (5). The minimum and maximum values of the obtained temperatures are estimates of the lower and upper bounds $T_{l}$ and $T_{u}$. These bounds will be reported in this paper, together with their corresponding average spectral emissivity values, calculated using Equation (4) and denoted by $\varepsilon_{l}$ and $\varepsilon_{u}$.

The bounds were calculated in the MATLAB software environment (version R2013a, The MathWorks, Inc., Natick, MA, USA) using the Levenberg–Marquardt algorithm for performing the nonlinear least squares optimization [39,40]. This algorithm resulted in fast convergence, approaching a quadratic convergence rate and typically requiring five iterations to find the minimum. The minimization procedure requires the computation of a matrix inverse in each iteration, giving the problem a computational complexity of O($N^{3}$). Since the minimization needs to be performed for each measured spectrum, the complexity scales linearly with the number of spatial points.

A useful indicator for the validity of the results is the reduced chi-squared statistic:

(7) $$\chi^{2}=\frac{1}{N-2}\sum_{i=1}^{N}\frac{{\left|E_{i}-\left({\hat{B}}_{\varepsilon }-{\hat{A}}_{\varepsilon }\frac{\lambda_{i}-\lambda_{1}}{\lambda_{N}-\lambda_{1}}\right)B\;\left(\lambda_{i},\hat{T}\right)\right|}^{2}\;}{\sigma_{E_{i}}^{2}}.$$

Large values of $\chi^{2}$ correspond to large fitting errors, indicating that in those cases the emitted spectra cannot be fitted adequately using the linearly decreasing emissivity function given by Equation (3). If the emissivity variation with wavelength is perfectly linear, $\chi^{2}$ becomes equal to 1. The chi-squared statistics corresponding to the lower and upper temperature bounds will be denoted by $\chi_{l}^{2}$ and $\chi_{u}^{2}$, respectively.

## 3. Measurement System

In this section, the hyperspectral imaging system that was used for performing the measurements is presented. An analysis of the measurement uncertainty then reveals that the uncertainty is mostly determined by the optical sensitivity of the imaging system. The section ends with a short description of the experimental setup.

### 3.1. Hyperspectral Imaging

**Figure 1:**
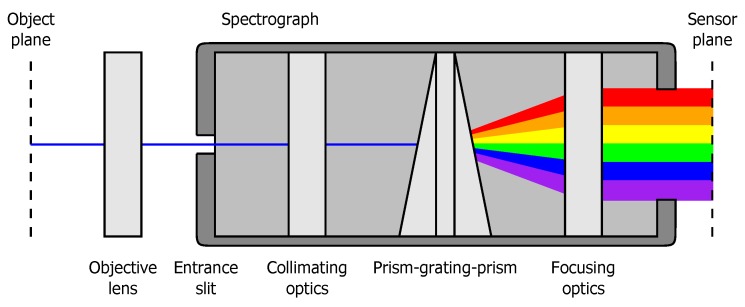
Schematic overview of the hyperspectral imaging system.

The measurements were performed with a SPECIM PFD V10E hyperspectral line camera (Oulu, Finland). The light captured by the objective lens passes through a narrow slit, after which it is dispersed by a diffraction grating and projected onto the camera sensor. A schematic overview of the hyperspectral imaging system is given in Figure 1. The resulting images contain the spectra of a large number of adjacent points on a line. This line has a thickness of 40 µm, a spatial resolution of 12 µm/pixel and counts 140 pixels, resulting in a total length of 1.7 mm. Each of the measured spectra contains information in the visible and near infrared (VNIR) region from 400 nm to 950 nm with a spectral resolution of 1.1 nm/pixel (N=512). The images were captured with a bit depth of 10-bits and an exposure time of 78 µs at a frame rate of 1000 frames per second.

**Figure 2:**
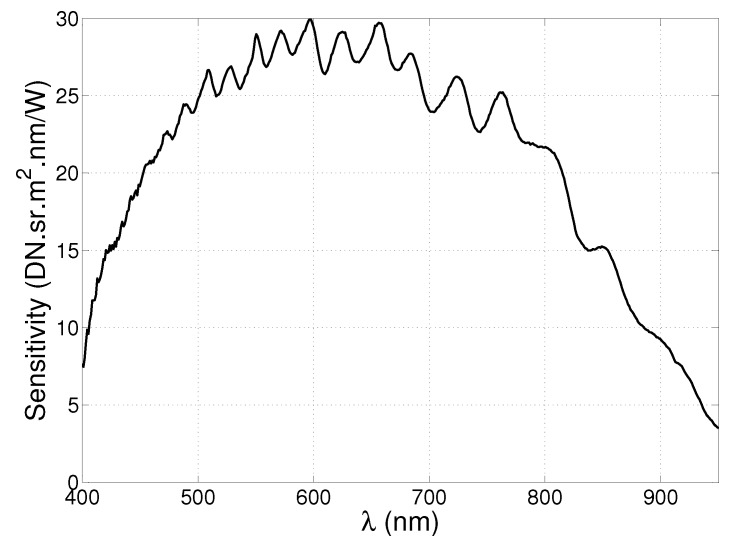
Measured sensitivity of the hyperspectral imaging system.

An absolute radiance calibration of the camera was performed by measuring the spectrum of an Optronic Laboratories OL455 integrating sphere calibration source with a halogen lamp (Orlando, FL, USA). The same spectrum was measured with a National Institute of Standards and Technology (NIST) traceable JETI Specbos 1211 spectroradiometer (Jena, Germany). The sensitivity of the imaging system is obtained as the ratio of these two spectra and can be used to correct the measurements made during the experiments. The sensitivity of the hyperspectral imaging system is plotted in Figure 2 as a function of wavelength. The ripple that can be seen in this plot is an interference pattern that results from the presence of different layers of material at the sensor surface. Denoting the sensitivity at wavelength $\lambda_{i}$ by $G_{i}$, and the measured value of the pixel corresponding to wavelength $\lambda_{i}$ by $D_{i}$, a measurement can be converted into an absolute radiance value as follows:

(8) $$E_{i}=\frac{D_{i}-D_{i}^{0}}{G_{i}}.$$

Here, $D_{i}^{0}$ represents the value reported by the pixel when no light is present. This is referred to as the dark value and is typically nonzero in digital sensor devices. The dark value can be measured by averaging a series of images that are captured with the lens closed.

### 3.2. Uncertainty Analysis

The uncertainty of $E_{i}$, denoted by $\sigma_{E_{i}}^{2}$, can be calculated from Equation (8) in a straightforward way:

(9) $$\sigma_{E_{i}}^{2}=\left[\frac{\sigma_{D_{i}}^{2}+\sigma_{D_{i}^{0}}^{2}}{{\left(D_{i}-D_{i}^{0}\right)}^{2}}+\frac{\sigma_{G_{i}}^{2}}{G_{i}^{2}}\right]E_{i}^{2}.$$

The uncertainties $\sigma_{D_{i}^{0}}^{2}$ and $\sigma_{G_{i}}^{2}$ are very low, as the dark values $D_{i}^{0}$ and the sensitivity function $G_{i}$ are obtained from many measurements that are averaged out. The measurement uncertainty is therefore approximately equal to:

(10) $$\sigma_{E_{i}}^{2}\approx \frac{\sigma_{D_{i}}^{2}}{G_{i}^{2}}.$$

It can be seen from Equation (10) that the uncertainty $\sigma_{E_{i}}^{2}$ at a specific wavelength is low if the optical sensitivity at that wavelength is high. The radiance measurements at these wavelengths will have the most influence on the calculated temperature estimates obtained with the least squares optimization procedure given by Equation (5).

The uncertainty of the temperature estimate obtained by solving Equation (5) can be calculated by defining the error function $e_{i}$ and the Jacobian matrix *J*:

(11) $$e_{i}=\frac{E_{i}-\left(B_{\varepsilon }-A_{\varepsilon }\frac{\lambda_{i}-\lambda_{1}}{\lambda_{N}-\lambda_{1}}\right)B\;\left(\lambda_{i},T\right)}{\sqrt{\sigma_{E_{i}}^{2}}},$$

(12) $$J=\left(\begin{array}{cc} \frac{\partial e_{1}}{\partial T} & \frac{\partial e_{1}}{\partial B_{\varepsilon }}\\ \vdots & \vdots \\ \frac{\partial e_{N}}{\partial T} & \frac{\partial e_{N}}{\partial B_{\varepsilon }} \end{array}\right).$$

The uncertainty of the temperature estimate can then be found as the first diagonal element of the error covariance matrix *C*:

(13) $$C={\left.{\left(J^{T}J\right)}^{-1}\right|}_{{\hat{A}}_{\varepsilon },{\hat{B}}_{\varepsilon },\hat{T}}=\left(\begin{array}{cc} \sigma_{\hat{T}}^{2} & \sigma_{{\hat{B}}_{\varepsilon }\hat{T}}\\ \sigma_{{\hat{B}}_{\varepsilon }\hat{T}} & \sigma_{{\hat{B}}_{\varepsilon }}^{2} \end{array}\right).$$

Note that the uncertainty of ${\hat{A}}_{\varepsilon }$ is zero since we have assigned it a fixed value as explained in Section 2. Using Equation (4), the uncertainty of the average spectral emissivity estimate $\hat{\varepsilon }$ is found as $\sigma_{\hat{\varepsilon }}^{2}=\sigma_{{\hat{B}}_{\varepsilon }}^{2}$.

### 3.3. Experimental Setup

**Figure 3:**
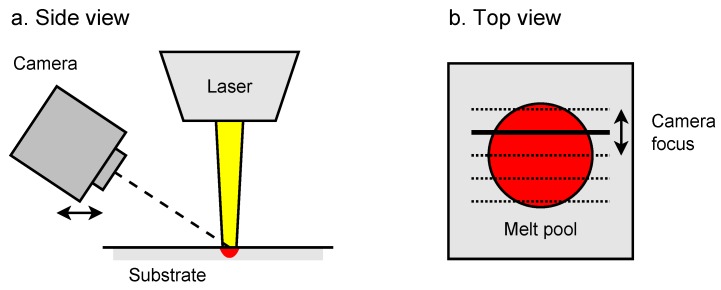
Side view (**a**) and top view (**b**) of the measurement setup.

A laser melting experiment was performed by focusing an IPG YLS-1000 fiber laser (Oxford, MA, USA) with a center wavelength of 1070 ± 10 nm on a 20 mm thick substrate of AISI 316L stainless steel. This resulted in the creation of a melt pool that maintained a stable size after a few seconds of irradiation. A flow of argon gas was directed towards the substrate in order to minimize oxidation of the melt pool surface. A flat top laser beam was used with a diameter of 1200 µm in the focus plane and with a total laser power of 250 W. Due to the presence of the laser head, the camera could not be positioned perfectly normal to the melt pool surface and was positioned at an angle of 38 degrees relative to the surface normal. Measurements were made at different locations by horizontally displacing the camera so that a complete temperature profile of the melt pool surface could be obtained. A schematic overview of the measurement setup is given in Figure 3.

## 4. Results and Discussion

The analysis of the experimental results starts with a microscope inspection of the part after the surface was irradiated with the laser beam. Different zones corresponding to a fully liquid, fully solid and a mushy region can be identified in the microscope image. Their locations are compared to the on-line temperature measurements made with the hyperspectral camera setup, and the measurements of the temperature and emissivity in each of these regions are then discussed.

### 4.1. Microscope Inspection

**Figure 4:**
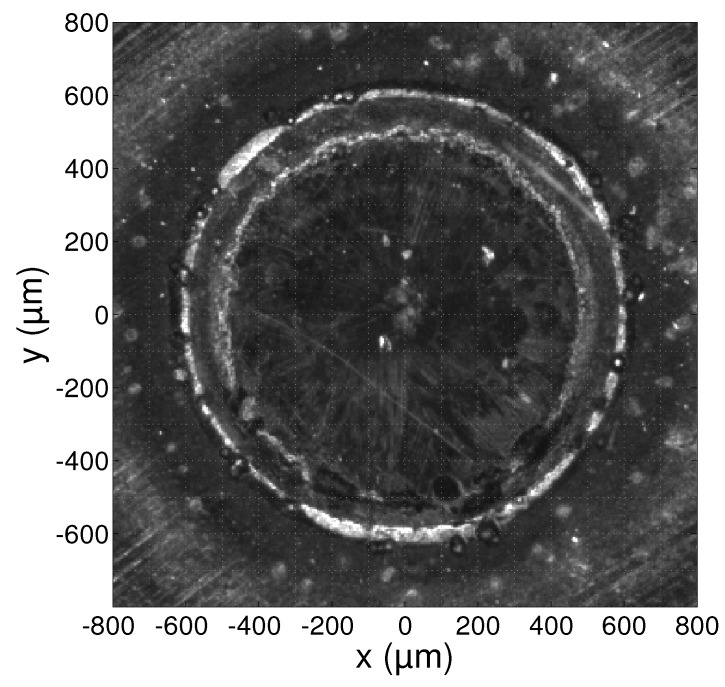
Microscope image of the substrate surface after irradiation with the laser beam.

A microscope image of the substrate surface taken after irradiation with the laser beam is shown in Figure 4. The image was taken with a resolution of 5 µm/pixel. The outer circle that can be seen in the image has a diameter of 1200 µm and encloses the area that was irradiated by the laser beam. The smaller inner circle has a diameter of ca. 960 µm and marks the region that was in a fully liquid state during the experiment, i.e., the melt pool. The small region between these two circles is called the mushy region [41] and contains the material that was in a partially solid and liquid state.

### 4.2. Temperature and Emissivity Measurements

**Figure 5:**
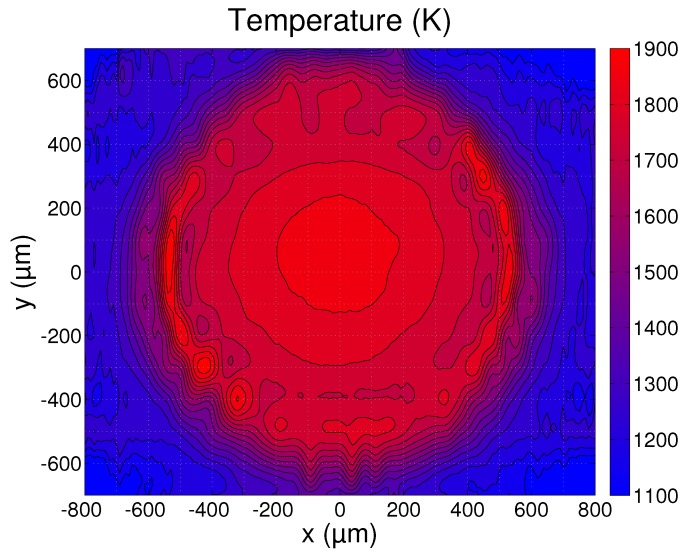
Measured surface temperature distribution during laser melting. The contours mark the isotherms from 1100 K to 1900 K in steps of 50 K.

While the substrate was being irradiated with the laser beam, measurements of the emitted light were used to calculate a temperature profile according to the procedure outlined in Section 2. The MATLAB software was used to perform the nonlinear least squares calculations. The temperature profiles were estimated from 100 consecutive images and time averaged. A single profile contains high resolution (12 µm/pixel) temperature information along the *x*-axis that is defined in Figure 4. The camera focus location was then displaced along the *y*-axis in 100 µm increments and new measurements were made at each location. A contour plot of the combined surface temperature profiles is presented in Figure 5. The plotted temperature profiles are the averages of the estimated lower and upper bounds $T_{l}$ and $T_{u}$ and the profiles at intermediary *y* locations were determined using cubic interpolation. The contours mark the isotherms from 1100 K to 1900 K with a spacing of 50 K.

Due to the finite thickness of the slit that is used in the hyperspectral camera, the measured temperature profiles represent a combination of the temperatures in a region of 40 µm along the *y*-axis. As a result, small features such as the bright circle with 1000 µm diameter can only be seen in the measurements at |y|<300 µm and are smeared out at the other locations. The temperature profile measured at y=0, therefore, contains the most detailed temperature information and will be discussed further in the following sections.

**Figure 6:**
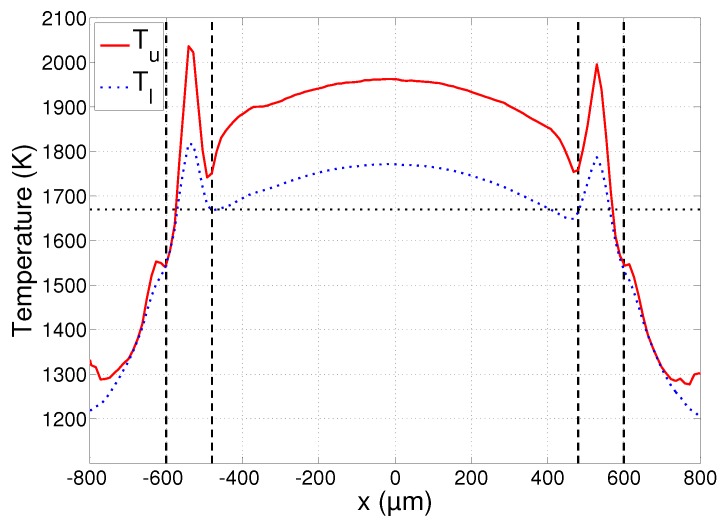
Temperature profile at *y* = 0 during laser melting. The solid curve corresponds to the upper temperature bound and the dotted curve to the lower temperature bound. The dashed vertical lines separate the liquid, mushy and solid regions. The dotted horizontal line indicates the melting point.

A plot of the temperature profile at y=0 is shown in Figure 6. The solid curve corresponds to the estimates of the upper temperature bound $T_{u}$ and the dotted curve to those of the lower bound $T_{l}$. As explained in Section 3.2, the uncertainties of these bounds can be calculated using Equation (13). Due to the use of a large number of wavelengths in the fitting process (N=512), the obtained uncertainties are very low. In the region |x|<600 µm, the signal strength of the measurements is also high and we have an uncertainty as low as $\sigma_{T_{l}}<1.4\;K$ and $\sigma_{T_{u}}<4.0\;K$. The signal strength at |x|>600 µm is lower and the uncertainty quickly increases. In the region 600 µm<|x|<800 µm, it still remains acceptably low at $\sigma_{T_{l}}<20\;K$ and $\sigma_{T_{u}}\;<\;80\;K$. It must be stressed that these uncertainties only give an indication of the error on the temperature estimate provided that the underlying emissivity model is perfect. The uncertainties therefore only give an idea of the region in which the true temperature bounds $T_{l}$ and $T_{u}$ are located.

**Figure 7:**
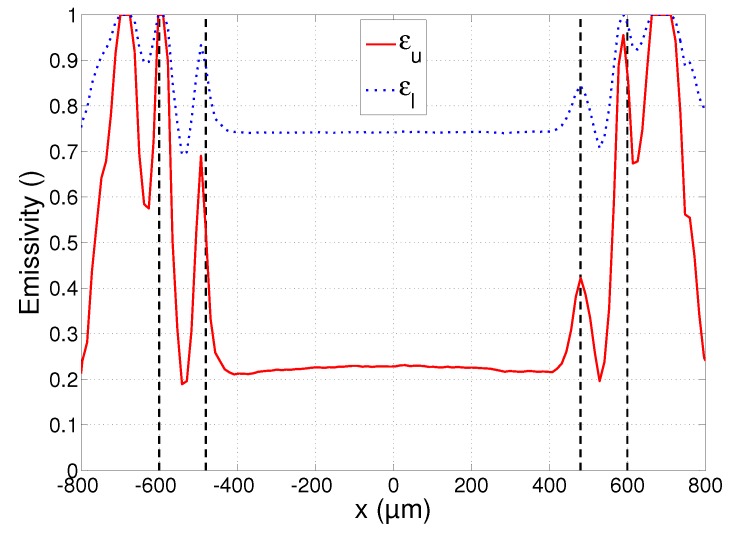
Emissivity profile at *y* = 0 during laser melting. The solid curve corresponds to the upper temperature bound (lowest emissivity) and the dotted curve to the lower temperature bound (highest emissivity). The dashed vertical lines separate the liquid, mushy and solid regions.

The profiles of the estimated lower and upper emissivity bounds $\varepsilon_{l}$ and $\varepsilon_{u}$ are plotted in Figure 7. The emissivity estimation errors are larger than those of the temperatures. They are again smallest in the region |x|<600 µm, where $\sigma_{\varepsilon_{l}}<0.007$ and $\sigma_{\varepsilon_{u}}<0.030$, and larger at 600 µm<|x|<800 µm, where $\sigma_{\varepsilon_{l}}<0.08$ and $\sigma_{\varepsilon_{u}}<0.25$.

**Figure 8:**
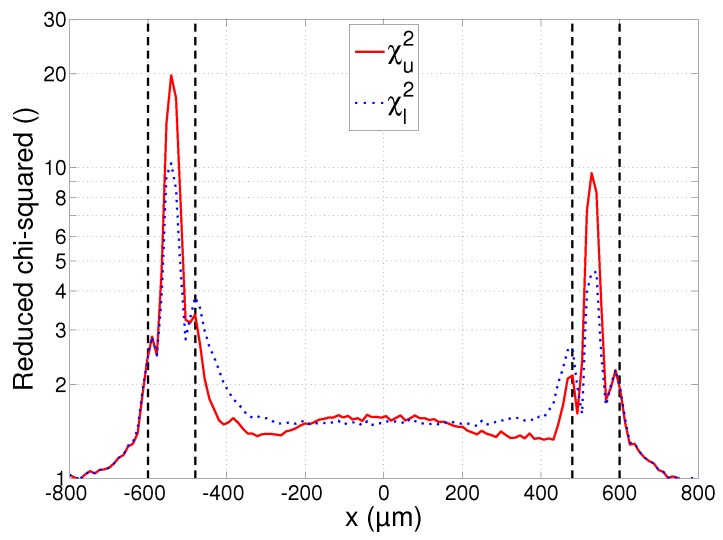
Reduced chi-squared profile at *y* = 0 during laser melting. The solid curve corresponds to the upper temperature bound and the dotted curve to the lower temperature bound. The dashed vertical lines separate the liquid, mushy and solid regions.

A final plot that will be used in the subsequent discussions is that of the chi-squared statistics $\chi_{l}^{2}$ and $\chi_{u}^{2}$, calculated using Equation (7). The plot is shown in Figure 8 and will provide an indication of the reliability of the estimates. It must be remarked that the small asymmetry that can be noticed in all the plots of Figure 6, Figure 7 and Figure 8 is due to the imperfect alignment of the camera with the substrate surface and resulted in the right parts of the images being slightly more out of focus than the left parts.

The three regions identified in Section 4.1—liquid, mushy and solid region—are separated from each other on the plots of Figure 6, Figure 7 and Figure 8 by the dashed vertical lines. Each of these regions will now be discussed in more detail.

### 4.3. Liquid Region

The main parts of the captured images consist of measurements of the melt pool surface, where the material is in a fully liquid state. The plot of Figure 6 shows that the temperature is highest at the center of the melt pool and gradually decreases towards the edges, as expected. The peak temperature is in good correspondence with the values reported in the literature for similarly sized melt pools, obtained using standard pyrometric techniques with a grey body assumption [9,22,23]. The reported values are situated between 1800 K and 2000 K and fall within the interval marked by the lower and upper temperature bounds $T_{l}$ and $T_{u}$ shown in Figure 6. Due to the low uncertainty values obtained in this region, the temperature bounds can be determined with high precision. The difference between these bounds is ca. 200 K in the entire melt pool, showing that the true temperature can be determined with an accuracy of roughly 10%.

The accuracy of the associated average spectral emissivity values is much lower, as can be seen in Figure 7. The possible values that the emissivity can assume range from 0.20 up to 0.75. The high values in this range seem unlikely, as typical normal spectral emissivity values reported for polished stainless steel in the VNIR range are around 0.3–0.5 [35,36,37,38]. Another interesting conclusion that can be made from this plot is that the emissivity seems to be almost constant inside the melt pool, showing no significant dependence on temperature.

The fact that the linearly decreasing emissivity model given by Equation (3) can be used to reliably fit the measured spectra is illustrated in Figure 8. The value of the reduced chi-squared statistic is approximately equal to 1.5, which is very close to the optimal value of 1. This means that successful fits were obtained with small residual errors. The plots also show that there is no significant difference between the residual errors of the upper and lower bounds, even though these bounds were calculated with two very different average spectral emissivity values. This is an important observation, as it means that the measurements can be fitted almost equally well with an arbitrary average spectral emissivity value, as long as the slope of the emissivity decrease with wavelength is adjusted accordingly. The constraints imposed on the emissivity ($0<\varepsilon_{\lambda }\leq 1$ and a decreasing slope) allow us to put bounds on these values, but the true value of the emissivity cannot be determined from the measurements alone.

### 4.4. Mushy Region

While the spectra emitted by the material in its liquid state seem to comply with the emissivity model of Equation (3), the material in the mushy region presents a completely different situation. The fitting error plotted in Figure 8 increases exponentially in this region up to a value of ca. 10, which is an order of magnitude higher than in the liquid region. This is true for both the upper and lower bounds, showing that the spectra cannot reliably be fitted to a black body spectrum multiplied by a spectral emissivity value that decreases linearly with wavelength. While not shown on these plots, no better fit could be obtained by also allowing the spectral emissivity to increase with wavelength. This leads to the conclusion that the material in the mushy region does not exhibit a clear linear emissivity variation with wavelength and that the temperatures cannot be determined reliably using the emissivity model of Equation (3). The apparent rise of the temperature in the mushy region of Figure 6 is a result of this model mismatch and is clearly incorrect.

While the measurements may not contain reliable quantitative information about the temperature in this region, the emissivity plot of Figure 7 still presents some useful information: the spatial boundaries of the mushy region can clearly be identified as the emissivity peaks at |x|=480 µm and |x|=600µm. This is in correspondence with the mushy region boundaries identified in the microscope image in Figure 4. The temperature estimates at these boundaries are approximately 1550 K and 1700 K. This interval correctly includes the melting point of AISI 316L stainless steel, which is approximately 1670 K [42]. The melting point is indicated on the plot of Figure 6 as the dotted horizontal line.

### 4.5. Solid Region

Outside the region irradiated by the laser beam, the material remained in a fully solid state. Moving away from the melt pool, the temperature decreases and the signal measured by the camera quickly vanishes. The good fit indicated by the reduced chi-squared statistic reaching a value of 1 near |x|=800 µm in Figure 8 is a result of this very low signal: a spectrum that is zero everywhere can be fitted with any temperature and/or emissivity value. The estimates at 600 µm<|x|<800 µm still have a chi-squared value larger than 1 and convey some useful information, showing that the temperature indeed decreases quickly, as does the emissivity. The high values of the average spectral emissivity can be explained by the large amount of oxidation that is present around the irradiated zone of the substrate. It can be seen in the microscope image of Figure 4 that the density of the oxidation layer decreases gradually when moving away from the irradiated zone. This is visible in the plot of Figure 7 as a gradual decrease of the average spectral emissivity. The measurements at |x|>800 µm are not included in the plots as the reduced chi-squared statistic remains 1 in this region, and the temperature and emissivity estimates fluctuate randomly.

If more reliable estimates of the temperature in the solid region are desired, the signal-to-noise ratio in this region must be increased. One way of doing this is by increasing the exposure time when capturing the images. However, this makes the images more prone to motion blur and possibly results in saturation of the high temperature parts of the images. The latter may influence the measured signal in the low temperature parts of the images due to the limited resolving power of the optical system. A better solution is to perform the measurements with a device that can capture the emitted spectral radiance at larger wavelengths, i.e., in the infrared region. While this would indeed allow a better measurement of the temperature profile in the solid region, it would, in turn, be a less suitable and more expensive solution for the high temperature measurements in the liquid region.

## 5. Conclusions

This paper presents a method for measuring the surface temperature distribution of a melt pool of AISI 316L stainless steel with high spatial resolution. The spectral radiance emitted by the melt pool surface is captured with a hyperspectral camera and used in a nonlinear least squares optimization routine for obtaining an estimated temperature value. The spectral emissivity is assumed to decrease linearly with wavelength, allowing the computation of the lower and upper bounds of a temperature interval that includes the true temperature. The presented system is sensitive in the VNIR region and was used to measure the temperature distribution of the melt pool surface with a spatial resolution of 12 µm/pixel.

Inspection of a microscope image of the substrate surface taken after laser irradiation allows a subdivision of the image into a fully liquid, fully solid and a mushy region. These regions can also be identified in the emissivity profiles estimated from the hyperspectral images. The results show that the temperature in the liquid region can be determined with an accuracy of ca. 10%. To the best of the authors’ knowledge, an absolute temperature profile of the liquid melt pool during laser melting with high spatial resolution has not been previously reported in the literature. On the other hand, no quantitative information about the temperature distribution in the mushy region could be obtained, as the emissivity does not vary linearly with wavelength in this region. While the system was designed for high temperature measurements, some information about the low temperature solid region was also acquired. If more accurate low temperature information is desired, the use of a hyperspectral imaging system that is sensitive to larger wavelengths should be considered.

The presented hyperspectral imaging system can be used as a tool for monitoring the temperature distribution during processes with large local temperature variations, such as laser welding and laser cladding. As it was also shown that the melt pool boundary can be determined with high accuracy, it is an excellent candidate sensor for a melt pool control system in applications where high geometrical accuracy is required, e.g., in the field of additive manufacturing.
